# Addressing scalability and managing sparsity and dropout events in single-cell representation identification with ZIGACL

**DOI:** 10.1093/bib/bbae703

**Published:** 2025-01-07

**Authors:** Mingguang Shi, Xuefeng Li

**Affiliations:** School of Electrical Engineering and Automation, Hefei University of Technology, Hefei, Anhui, China; School of Electrical Engineering and Automation, Hefei University of Technology, Hefei, Anhui, China

**Keywords:** single-cell representations, zero-inflated graph attention collaborative learning, a zero-inflated negative binomial model, co-supervised learning

## Abstract

Despite significant advancements in single-cell representation learning, scalability and managing sparsity and dropout events continue to challenge the field as scRNA-seq datasets expand. While current computational tools struggle to maintain both efficiency and accuracy, the accurate connection of these dropout events to specific biological functions usually requires additional, complex experiments, often hampered by potential inaccuracies in cell-type annotation. To tackle these challenges, the Zero-Inflated Graph Attention Collaborative Learning (ZIGACL) method has been developed. This innovative approach combines a Zero-Inflated Negative Binomial model with a Graph Attention Network, leveraging mutual information from neighboring cells to enhance dimensionality reduction and apply dynamic adjustments to the learning process through a co-supervised deep graph clustering model. ZIGACL’s integration of denoising and topological embedding significantly improves clustering accuracy and ensures similar cells are grouped closely in the latent space. Comparative analyses across nine real scRNA-seq datasets have shown that ZIGACL significantly enhances single-cell data analysis by offering superior clustering performance and improved stability in cell representations, effectively addressing scalability and managing sparsity and dropout events, thereby advancing our understanding of cellular heterogeneity.

## Introduction

Single-cell representations learning involves creating numerical or graphical depictions of individual cells’ properties derived from high-throughput data, such as single-cell RNA sequencing (scRNA-seq) [1]. These representations are crucial for understanding cellular heterogeneity and the unique functional roles of each cell in a tissue or organism. To identify single-cell representation, the process involves classifying and characterizing individual cells based on their gene expression profiles. This critical aspect of single-cell transcriptomics enables researchers to dissect the complexities of biological systems at the resolution of individual cells. The challenge in identifying single-cell representation arises from the nature of the data, which is high-dimensional, sparse, and noisy [2]. Each cell is represented by the expression levels of thousands of genes, many of which may exhibit low or undetectable expression due to technical limitations known as dropout events. Furthermore, the biological variability among cells requires sophisticated computational techniques to identify significant patterns in the single-cell data.

Traditional identification methods for single-cell representations, like manual or semi-automated analysis with basic statistics, are effective yet limited by their reliance on predefined markers and their failure to capture transcriptome-level heterogeneity [3]. The field of single-cell analysis has witnessed transformative advancements with the development of high-throughput sequencing technologies, particularly scRNA-seq. Computational methods have evolved in parallel to address the aforementioned challenges presented by the massive and complex datasets generated by scRNA-seq. Manifold learning methods like t-SNE [4] and UMAP [5] simplify high-dimensional data into two or three dimensions, preserving key structures. Clustering algorithms including k-means, hierarchical, and graph-based methods, identify novel cell types and states without prior markers [6]. Moreover, both supervised and unsupervised machine learning are increasingly used to predict cell types, states, and lineage trajectories from scRNA-seq data [7, 8].

Recent advancements in neural network approaches have significantly enhanced the representation of cells in single-cell data analysis. [9–13]. These methods leverage increasingly sophisticated algorithms to more accurately interpret the vast amount of data generated by technologies like scRNA-seq. Innovations in dimensionality reduction, clustering, and machine learning, including deep learning, have enhanced our ability to distinguish cell types, states, and developmental trajectories, providing deeper insights into cellular diversity and function. These improvements help in visualizing complex data, identifying novel cell phenotypes, and predicting cellular behavior with greater precision, thus expanding our understanding of biological processes at the single-cell level.

Despite advancements in the field of single-cell representation, significant challenges in research persist, particularly concerning the scalability of current methods. As the volume of scRNA-seq datasets increases, the efficiency and accuracy of existing computational tools often diminish. This underscores the necessity for developing algorithms that can efficiently process millions of cells while retaining detailed analytical specificity. Additionally, a significant hurdle is reliably associating dropout events with specific biological functions, which generally necessitates complex supplementary experiments. These efforts are frequently complicated by the potential inaccuracies inherent in annotating cell types.

This work introduces a groundbreaking clustering method for scRNA-seq data, termed the Zero-Inflated Graph Attention Collaborative Learning (ZIGACL) method. ZIGACL employs a dual-strategy approach: it incorporates a zero-inflated negative binomial (ZINB) model [14] to address dropout events, and a Graph Attention Network (GAT) [15] that leverages information from neighboring data points for efficient dimensionality reduction. Subsequently, a co-supervised mechanism refines the deep graph clustering model. By utilizing a constructed graph, the model applies GATs under a co-supervised learning paradigm, dynamically adjusting attention across the graph based on the learning objectives. Through the synergistic integration of denoising processes and topological embedding, ZIGACL effectively generates cell representations that enhance clustering, ensuring that similar cells are proximal in the latent space. Comparative analyses conducted with ZIGACL across nine real scRNA-seq datasets demonstrate its superior clustering efficacy, significantly advancing the analysis of single-cell data. ZIGACL also facilitates the development of more stable cell representations, highlighting its contribution to the field. Additionally, it effectively addresses challenges of scalability and manages sparsity and dropout events in data processing.

## Results

### An overview of ZIGACL

**Figure 1:**
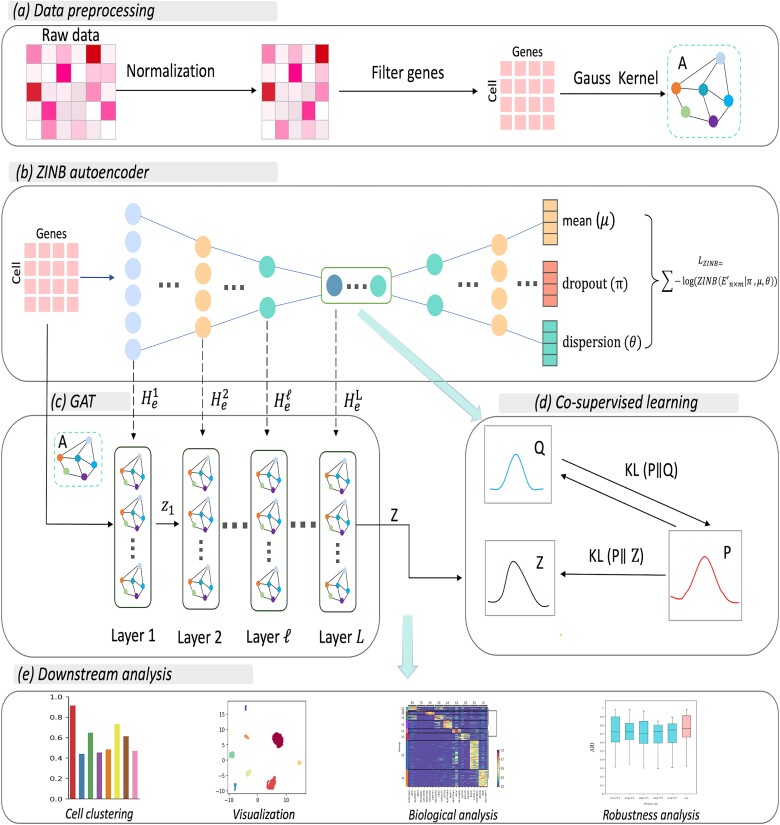
A graphical illustration of the ZIGACL model. (a) the single-cell data were normalized and scaled to correct for sequencing depth and cell-specific variations. The top genes showing the highest variability, measured by dispersion coefficient, were selected for their biological importance. An adjacency matrix was created using a gaussian kernel between cells to determine their similarity. (b) ZINB-based autoencoder is designed to handle the sparsity in scRNA-seq data through zero inflation and negative binomial distribution, enabling more accurate modeling of gene expression levels in individual cells. (c) GAT utilizes attention mechanisms to weigh the importance of neighboring cells in the gene expression data. This approach helps in capturing the complex cellular interactions and enhances the interpretability of cellular relationships. (d) Co-supervised learning utilizes the clustering loss derived from both the autoencoder and GAT module as supervisory signals, enabling effective model refinements. This strategic approach addresses the challenge of inadequate structural information within the generated representations, thereby enhancing the accuracy of cell clustering and subsequent analyses. These distributions P, Q, and Z provide mutual supervision within a unified framework, enabling efficient end-to-end network training and learning. (e) Downstream analysis of scRNA-seq data encompasses identifying distinct cell clusters, enabling subsequent analyses like cell visualization and biological interpretation.

ZIGACL is specifically designed for single-cell analysis using scRNA-seq data, utilizing advanced algorithms to delve deeply into gene expression patterns. It includes three main modules: a ZINB-based autoencoder, a GAT, and a co-supervised learning method (Figure 1). The processing begins with scRNA-seq data preprocessing, followed by the ZINB autoencoder, which reduces gene expression data into a lower-dimensional space for analysis. The autoencoder has fully connected layers for both encoding and decoding, facilitating the learning of embedded scRNA-seq data features. During decoding, the ZINB distribution models data sparsity and overdispersion through three activation layers that estimate the ZINB parameters μ、θ and π, capturing scRNA-seq data’s statistical properties. An adjacency matrix, created using a Gaussian kernel, is input into the GAT to analyze cellular structural interrelations. The autoencoder’s encoded features are integrated with GAT to enhance cellular dynamics understanding. In the next phase, co-supervised learning refines the deep graph clustering model through three distribution models: target, clustering, and probability distributions. The target distribution P directs the training by capturing cell similarities or distances, while the clustering distribution Q iteratively refines to reflect the data’s clustering structure. The probability distribution Z focuses on enhancing cluster membership indicators in the latent space. Clustering algorithms like k-means categorize cells into groups based on gene expression profiles, identifying different cell types or states. Differential expression analysis across clusters highlights functional disparities by identifying significantly regulated genes. Dimensionality reduction methods, such as UMAP, simplify high-dimensional data for easier visual analysis and cluster identification.

The ZINB-based autoencoder network includes a Gaussian noise layer and two fully connected layers, transforming input to 256 and then 64 dimensions, each followed by batch normalization, to efficiently process and represent cellular data. The latent space is represented by a layer that reduces the 64-dimensional representation to 16 dimensions. The decoder mirrors the encoder, with two fully connected layers that transform from 16 to 64 dimensions and from 64 to 256 dimensions, respectively. Throughout the training L_ZINB phase and fine-tuning L phase, optimization is performed using the Adam optimizer. The learning rate is set as 0.001. Furthermore, to mitigate the risk of gradient explosion, a gradient clipping strategy is employed, limiting the L2 norm to a maximum of 3. An early stopping criterion is applied during fine-tuning: if the proportion of label changes falls below 0.1% of the total labels, training halts to prevent overfitting. ZIGACL is implemented in Python 3.8.

ZIGACL improves cell clustering by learning cell representations adaptable to various scales of single-cell data.

We evaluated ZIGACL across nine scRNA-seq datasets, as outlined in Table 1. These datasets, sourced from existing literature and carefully annotated with ground truth labels, serve as a rigorous benchmark with different scales for comparing ZIGACL against seven deep learning methods: scGNN [16], DESC [17], SC3 [18], Leiden [19], scDeepCluster [20], graph-sc [21], and Scanpy [22]. Each method was executed five times under default settings, and their performance was assessed based on the average values of two metrics: the Adjusted Rand Index (ARI) and Normalized Mutual Information (NMI). Both metrics gauge the accuracy of clustering results, with higher values indicating more precise clustering.

**Table 1:** Single-cell RNA sequencing (scRNA-seq) datasets representing a comprehensive view of gene expression for ZIGACL development and the identification of single-cell representations within a tissue.

Dataset	Cell number	Gene number	Cell types	Platform
Muraro	2122	19,049	9	CEL-seq2
Romanov	2881	21,143	7	ILLumina HiSeq
Klein	2717	24,175	5	ILLumina HiSeq
Qx_Bladder	2500	23,341	4	10 X Genomics
Qx_Limb_Muscle	3909	23,341	6	10 X Genomics
Qx_Spleen	9552	23,341	5	10 X Genomics
QS-seq2_Diaphragm	870	23,341	5	Smart-seq2
QS-seq2_Limb_Muscle	1090	23,341	6	Smart-seq2
QS-seq2_Lung	1676	23,341	11	Smart-seq2

**Figure 2:**
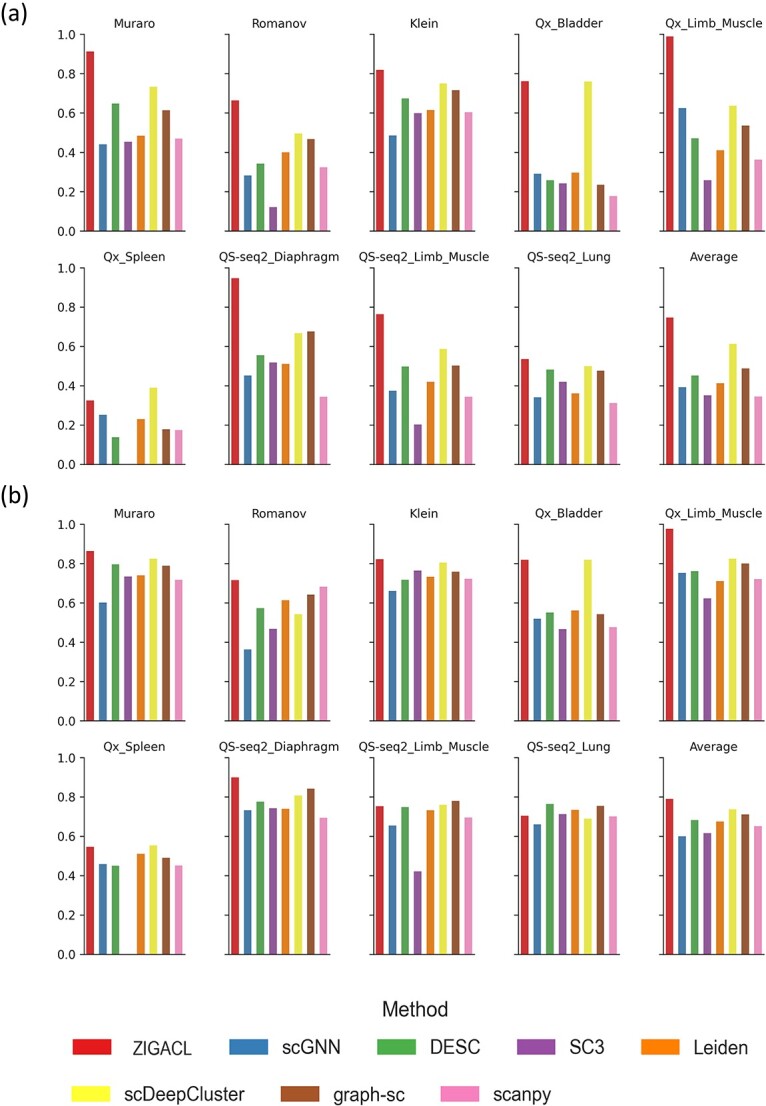
Comparative analysis of ZIGACL and baseline methods on scRNA-seq datasets using ARI and NMI metrics. This study conducts a comparative analysis of the ZIGACL clustering algorithm against baseline methods across nine scRNA-seq datasets. Performance is evaluated based on two statistical metrics: (a) the ARI and (b) NMI scores. ARI provides a measure of the similarity between the actual data groupings and those predicted by clustering algorithms, adjusting for chance alignment. NMI, meanwhile, quantifies the amount of shared information between predicted clusters and true labels, normalized to account for differences in dataset size and composition.

Figure 2a displays the ARI of eight methods across different datasets. In the Muraro dataset, ZIGACL scored an ARI of 0.912, achieving a 24.42% improvement over scDeepCluster and 107.27% over scGNN. For the Romanov dataset, ZIGACL’s ARI was 0.663, outperforming scDeepCluster and SC3 by 33.94% and 447.93%, respectively. In the Klein dataset, ZIGACL registered an ARI of 0.819, 9.20% and 68.87% better than scDeepCluster and scGNN. The Qx_Bladder dataset saw ZIGACL with an ARI of 0.762, marginally above scDeepCluster by 0.26% and surpassing scanpy by 330.51%. For the Qx_Limb_Muscle, ZIGACL reached an ARI of 0.989, outdoing scDeepCluster and SC3 by 55.5% and 284.82%. In the Qx_Spleen dataset, ZIGACL achieved the second-highest ARI of 0.325, which was 135.51% above DESC. The QS-seq2_Diaphragm dataset saw ZIGACL’s ARI at 0.947, which is 40.09% higher than graph-sc and 176.09% above scanpy. In the QS-seq2_Limb_Muscle dataset, ZIGACL recorded an ARI of 0.764, surpassing scDeepCluster and SC3 by 55.5% and 278.22%. Lastly, in the QS-seq2_Lung dataset, ZIGACL had an ARI of 0.535, outperforming scDeepCluster by 7.21% and scanpy by 71.47%. These figures underscore ZIGACL’s superior clustering accuracy and scalability across various scRNA-seq datasets.

In Fig. 2b, ZIGACL shows strong performance in the NMI metric across several datasets. For the Muraro dataset, it achieved an NMI of 0.864, surpassing scDeepCluster by 4.73% and scGNN by 43.52%. In the Romanov dataset, ZIGACL scored 0.715, outperforming scanpy and scGNN by 4.84% and 97.51%, respectively. In the Klein dataset, its NMI was 0.822, modestly above scDeepCluster by 2.24% and scGNN by 24.36%. For the Qx_Bladder dataset, ZIGACL tied with scDeepCluster at an NMI of 0.819 and exceeded SC3 by 75.37%. The Qx_Limb_Muscle dataset saw ZIGACL’s NMI at 0.977, outdoing scDeepCluster by 18.42% and SC3 by 56.82%. In the Qx_Spleen dataset, it achieved the second-highest NMI of 0.546, 21.30% above DESC. ZIGACL scored 0.899 in the QS-seq2_Diaphragm dataset, 6.77% higher than graph-sc and 29.35% over scanpy. In QS-seq2_Limb_Muscle, its NMI of 0.752 ranked third, 78.6% better than SC3. The QS-seq2_Lung dataset recorded an NMI of 0.703 for ZIGACL, 6.52% above scGNN. Overall, ZIGACL had the highest average scores in both ARI and NMI metrics across all datasets, with scores of 0.746 and 0.789, respectively. These are 21.7% and 7.2% higher than scDeepCluster and significantly better compared to the lowest-performing methods, with improvements of 116.23% in ARI and 31.5% in NMI. These results highlight ZIGACL’s ability to manage noise and sparsity in scRNA-seq data, enhancing its clustering accuracy and scalability across diverse single-cell datasets.

### ZIGACL enhances cell clustering and visualization in scRNA-seq data through handling sparsity and dropout events

**Figure 3:**
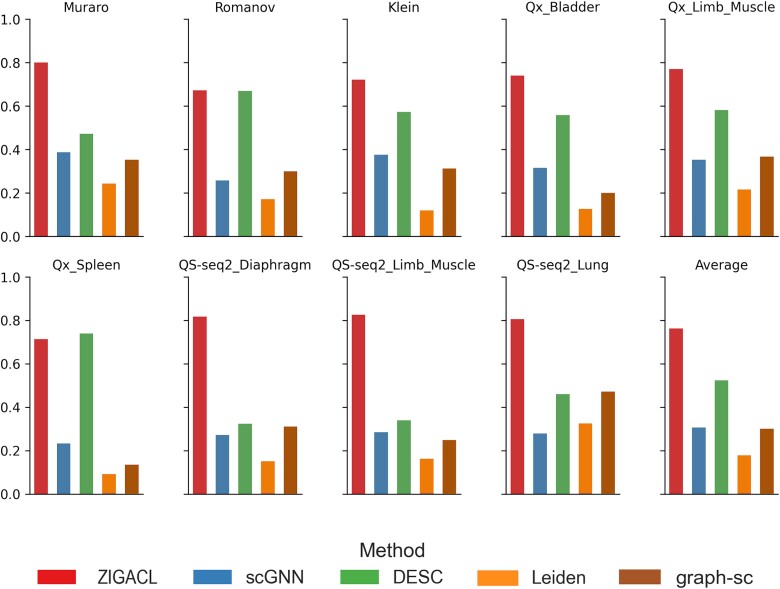
This analysis evaluates the clustering performance of the ZIGACL and four baseline methods across nine scRNA-seq datasets, using silhouette scores as the metric. Silhouette scores measure the cohesion and separation of clusters formed by each algorithm, providing insight into how effectively each method groups similar data points while keeping different groups apart.

To evaluate ZIGACL’s efficacy in learning optimized cell representations for clustering, we compared it against four established algorithms: scGNN, DESC, Leiden, and graph-sc. We used silhouette scores to assess intra-cluster homogeneity and inter-cluster heterogeneity, focusing on each algorithm’s ability to handle sparsity and dropout in scRNA-seq data. Figure 3 shows silhouette scores from various datasets, with ZIGACL consistently outperforming the others. In the Muraro dataset, ZIGACL’s average silhouette width (ASW) was 0.8, 69.49% higher than DESC and 229.22% above Leiden. In the Romanov dataset, its ASW of 0.672 marginally outdid DESC by 0.3% and significantly surpassed Leiden by 292.98%. ZIGACL’s ASW in the Klein dataset was 0.721, a 25.83% improvement over DESC and a 500.83% increase over Leiden. Its ASW in the Qx_Bladder dataset was 0.74, exceeding DESC by 32.38% and Leiden by 487.3%. In the Qx_Limb_Muscle dataset, ZIGACL’s ASW of 0.77 was 32.3% better than DESC and 256.48% above Leiden. Its ASW of 0.714 in the Qx_Spleen dataset ranked second, a 676.09% improvement over Leiden. In the QS-seq2_Diaphragm dataset, its ASW of 0.817 was 152.94% higher than DESC and 444.67% above Leiden. For QS-seq2_Limb_Muscle, ZIGACL’s ASW of 0.826 marked a 143.66% gain over DESC and 409.88% over Leiden. In the QS-seq2_Lung dataset, its ASW of 0.805 led graph-sc by 70.55% and exceeded scGNN by 189.57%. Overall, ZIGACL maintained the highest average ASW of 0.763 across datasets, 45.6% above DESC and 328.65% more effective than Leiden, highlighting its robust performance in clustering accuracy for single-cell analysis.

**Figure 4:**
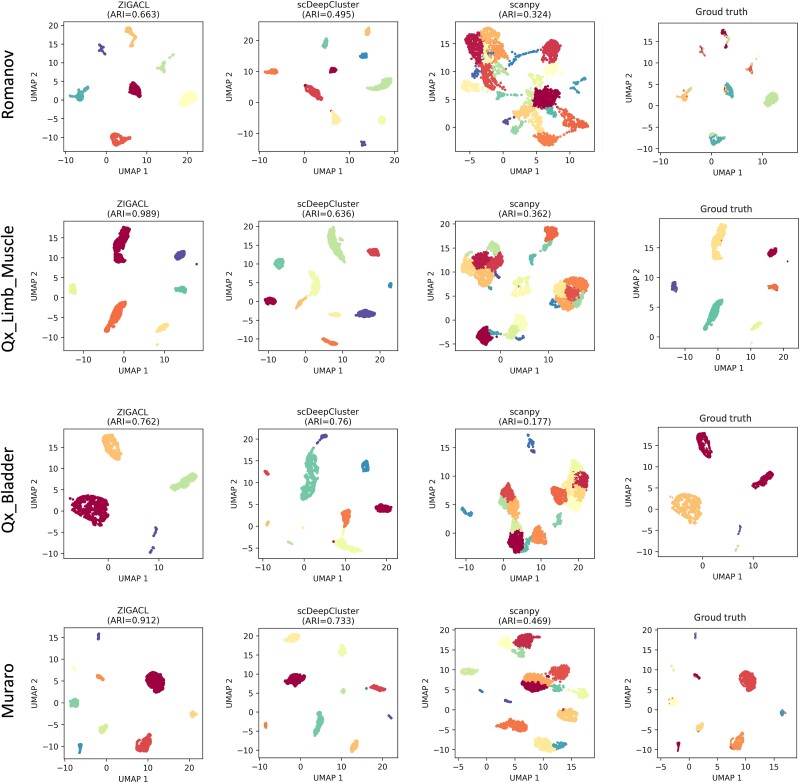
ZIGACL demonstrates superior performance compared to scDeepCluster and Scanpy in terms of cell clustering effectiveness across various datasets, as evidenced by visualization techniques. This suggests that ZIGACL is more adept at identifying and grouping cells with similar gene expression profiles, enhancing the clarity and utility of visual representations derived from complex scRNA-seq data.

To illustrate the intuitive clustering capabilities of ZIGACL, we employed it on four distinct single-cell datasets: Romanov, Qx_Limb_Muscle, Qx_Bladder, and Muraro, each characterized by different sample sizes and cellular subtypes. For cell visualization, as depicted in Fig. 4, we compared ZIGACL with scDeepCluster and Scanpy, utilizing ground truth labels for validation. This comparison was anchored in the use of UMAP to project preprocessed gene expression data onto a 2D space, thus facilitating an intuitive and visually accessible comparison. This approach not only underscores ZIGACL’s robustness in delineating cellular subpopulations but also highlights its efficiency in managing sparsity and dropout events, which are pivotal in preserving the fidelity of data interpretation in single-cell analyses. This dual focus on visual clarity and data integrity ensures that ZIGACL is academically rigorous in addressing the complexities of single-cell data clustering.

### Validating ZIGACL clustering and biological significance through cell annotation and marker gene analysis on the Muraro dataset

**Figure 5:**
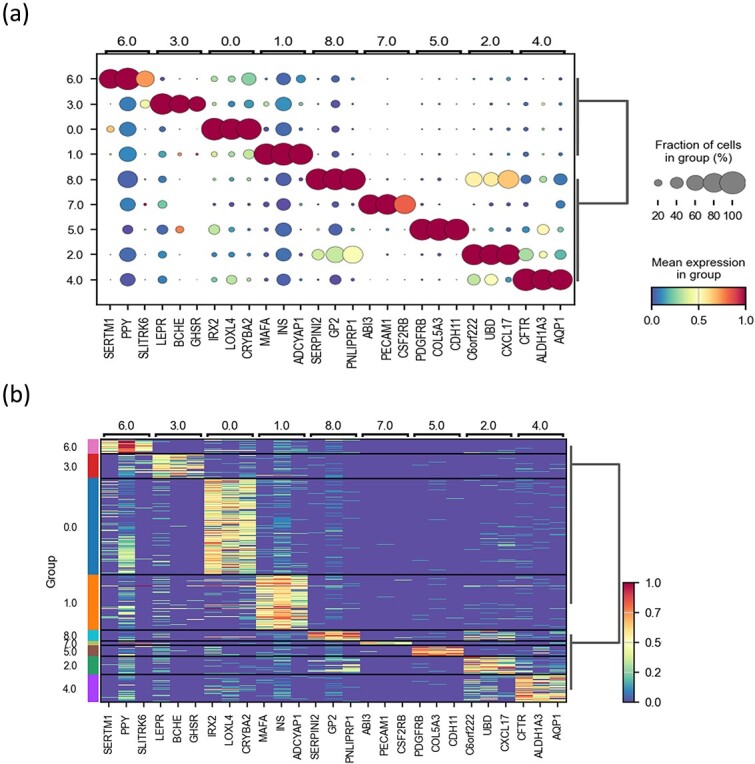
Visualization of marker gene expression using COSG in scRNA-seq data. The analysis features detailed visual representations of gene expression data, employing dot plots and heatmaps to illustrate the expression levels of the top three marker genes, as identified by the cluster of orthologous groups for each cluster. Figure 5a Presents dot plots that offer a granular view of the expression levels across different cells within the clusters. Meanwhile, Fig. 5b features heatmaps that provide a comparative overview of these expression patterns, allowing for easy observation of variations and similarities in gene activity across the clusters.

To validate the accuracy of ZIGACL-predicted clustering and explore their biological significance, we analyzed the Muraro dataset for cell annotation and marker gene identification. Using ZIGACL labels and ground truth, we aimed to identify differentially expressed genes (DEGs) per cluster for cell annotation. Initially, COSG (COSine similarity-based marker Gene identification) [23] was used to pinpoint marker genes for each predicted cluster, identifying the top 50 DEGs per cluster. We then calculated the overlap rate by comparing these DEGs with known genes of each cell type, finding common genes between the DEG sets of the predicted clusters and each cell type. The overlap rate was determined by dividing the intersection size by the reference set size. Clusters with the highest overlap rates were annotated as specific cell types. Expression dot plots were utilized, where dot color indicates average gene expression and size shows the proportion of cells expressing each gene (Fig. 5a). The analysis included 27 genes, highlighting the top three highly expressed genes from each cluster’s top 50. Based on the highest overlap rate of these genes, the clusters (0–1, 3–4, 6–8) were annotated as α cells, β cells, δ cells, ductal cells, γ cells, stromal cells, and acinar cells, respectively (Fig. 5b). To confirm if these marker genes match actual types, we compared them with genes in the CellMarker database (http://bio-bigdata.hrbmu.edu.cn/CellMarker/). The results indicate that the majority of these marker genes align with those listed in the database. Notably, three α-cell markers (IRX2, LOXL4, CRYBA2), identified using ZIGACL-predicted labels, are also recorded in the database. This indicates that ZIGACL’s accurate clustering enhances cell annotation and marker gene identification in scRNA-seq.

### Robustness analysis of ZIGACL

**Figure 6:**
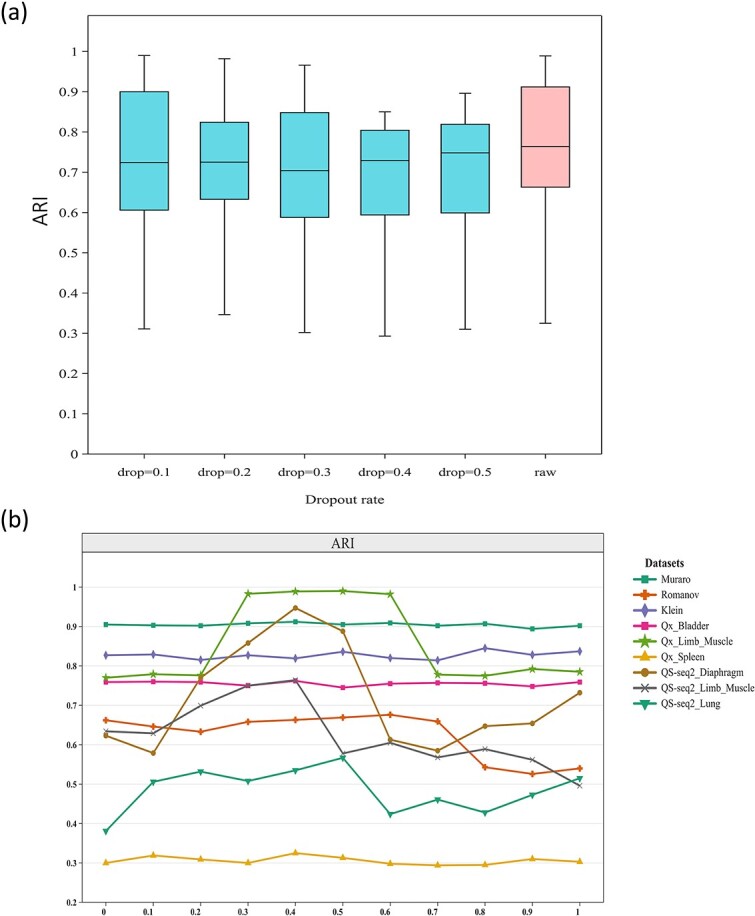
Model robustness validation. (a) Comparison of ARI values for ZIGACL on nine datasets under different dropout rates. It illustrates the ARI scores for the clustering outcomes at varying manual gene expression elimination rates within the scRNA-seq dataset, set at 10%, 20%, 30%, 40%, and 50%. Each experiment was replicated five times using distinct random seeds to ensure consistency. The baseline (raw refers to a dropout rate of 0) denotes ZIGACL’s performance prior to any manual data reduction. Results from the nine datasets are presented in each boxplot, where the median score is depicted by a solid line, and the whiskers denote the highest and lowest scores observed. (b) Comparison of clustering performance ARI on nine datasets by using of ZIGACL with different $\lambda$ parameters. The coefficient $\lambda$ was varied across a range of values $\left\{0,0.1,0.2,0.3,0.4,0.5,0.6,0.7,0.8,0.9,1.0\right\}$ to scrutinize its effect on performance.

To assess the ZIGACL model’s robustness, we conducted dropout experiments on nine real scRNA-seq datasets (Table 1). The dropout rate, defined as the proportion of missing expressed genes in the sequencing data, slightly reduced ARI values, but ZIGACL’s performance remained strong, demonstrating its resilience to incomplete data and its effectiveness in scRNA-seq data clustering (Fig. 6a). In the development of ZIGACL, we consider a dropout rate of 0 to yield the best performance based on the ARI. Comparing ZIGACL with other models under increasing dropout rates would significantly enhance our understanding of their stability in addressing similar challenges. To evaluate the robustness of the scDeepCluster model, we conducted dropout experiments on nine real scRNA-seq datasets, achieving an average ARI score of 0.613 across all datasets. The dropout experiments reveal notable fluctuations in average ARI scores at different rates. At a dropout rate of 0.1, the score is 0.581, increasing to 0.593 at 0.2. It continues to rise to 0.603 at a 0.3 dropout rate; however, there is a significant drop to 0.514 at 0.4. Although there is a slight recovery to 0.537 at a 0.5 dropout rate, the marked decrease from 0.3 to 0.4 underscores a critical instability in performance under increasing dropout conditions.

ZIGACL employs a balancing parameter, $\lambda$, to merge features from the ZINB denoising autoencoder and the GAT, enhancing the model’s ability to capture complex cellular relationships (Equation 16). A $\lambda$ setting of 0.4 proved optimal across all datasets, highlighting the effective integration of these components, which significantly boosts the model’s accuracy in identifying and categorizing complex cellular populations. Suboptimal performance is noted when $\lambda$ is at zero, but increases to 0.3 significantly improve clustering efficacy, emphasizing the critical role of the combined features from the autoencoder and graph attention layers. Different datasets showed varied responses to $\lambda$ adjustments; e.g. clustering performance in datasets like Qx_Limb_Muscle and QS-seq2_Limb_Muscle fluctuates notably with increasing $\lambda$, whereas it stabilizes in others once $\lambda$ exceeds 0.1 (Fig. 6b).

## Discussion

We introduced ZIGACL, a clustering model for scRNA-seq data that combines a graph attention network with a zero-inflated negative binomial autoencoder, effectively addressing challenges of scalability while managing sparsity and dropout events in data processing. Demonstrating superior performance, ZIGACL achieved higher ARI, NMI, and ASW metrics, excelling in delineating cell boundaries as illustrated in UMAP 2D representations. Additionally, the model showed proficiency in cell annotation and marker gene identification, exhibiting improved stability and robustness. These features underscore its biological relevance and highlight its potential to facilitate downstream analysis.

Deep learning methods, including SAUCIE [24], Scvis [25], BAVARIA [26], ScSemiGAN [27], scNym [28], and Cytoself [29], demonstrate significant potential for cell representation. These techniques manage high-dimensional, non-linear data to enable clustering, imputation, and embedding of single-cell datasets from scRNA-seq, CyTOF, and microscopy. Despite their high computational costs and risk of overfitting, these methods require extensive training data and careful tuning and validation to ensure accurate, meaningful representations. The ZIGACL method has been introduced to address significant challenges in the field of cell clustering and dimensionality reduction. This approach synergizes a ZINB model with a GAT. By harnessing the mutual information from neighboring cells, ZIGACL enhances the process of dimensionality reduction. It dynamically refines the learning process through a co-supervised deep graph clustering model, ensuring that both local and global cell interactions are effectively captured and utilized. A core innovation of ZIGACL lies in its integration of denoising and topological embedding techniques, which collectively enhance clustering accuracy. The method ensures that similar cells are clustered closely in the latent space, which is crucial for high-dimensional biological data where noise and variability can obscure true biological signals. The denoising aspect of ZIGACL filters out irrelevant variations, while the topological embedding preserves the intrinsic structure of the cell populations. This dual approach not only improves the reliability of clustering outcomes but also provides a more interpretable latent space where meaningful biological insights can be derived.

The ZINB autoencoder has emerged as a cutting-edge solution for tackling the inherent sparsity and dropout events in scRNA-seq data. It addresses excess zeros often caused by technical dropouts through a zero-inflated model, separating dropout events from true biological variation in gene expression. This crucial separation reduces biases and enhances true expression profiling. Additionally, its negative binomial component manages overdispersion in scRNA-seq data, where variance exceeds the mean. The ZINB autoencoder accurately fits the data, improving the analysis of cellular heterogeneity and gene dynamics. By integrating these models into an autoencoder, it supports dimensionality reduction and feature extraction, essential for clustering tasks.

ZIGACL specifically addresses these scalability challenges through several innovative strategies. By integrating a ZINB model with a GAT, ZIGACL leverages the strengths of both approaches while mitigating their respective limitations. The ZINB model helps manage the sparsity and dropout events typical in scRNA-seq data, improving data representation and clustering performance. The GAT component, while resource-intensive, is optimized within the ZIGACL framework to handle large-scale datasets more efficiently. ZIGACL incorporates mutual information from neighboring cells to enhance dimensionality reduction and applies dynamic adjustments through a co-supervised deep graph clustering model. These features contribute to improved efficiency and scalability, allowing ZIGACL to process large datasets effectively without compromising on clustering accuracy. While the effect of varying $\lambda$ on performance is discussed, the variation in response can likely be attributed to dataset-specific factors. For instance, datasets with higher complexity or noise levels may require greater denoising or more robust feature extraction via the GAT, making the balance between the two components more critical. In such cases, smaller values of $\lambda$ might underemphasize the contribution of the autoencoder, resulting in suboptimal performance. Conversely, for datasets with lower complexity or noise, a relatively small $\lambda$ may suffice, as the GAT and autoencoder components are already adequately balanced with a modest factor.

We benchmarked the performance of ASW on simulated scRNA-seq data, where cell types were known a priori. The simulated dataset was generated using the Splatter R package with default parameters, consisting of 5000 cells divided into five cell types. The proportion of cells within each type was randomly drawn from a uniform distribution, and the dataset comprised 10 000 genes. ASW values were calculated under varying dropout levels, showing a clear decreasing trend as dropout increased: an ASW of 0.762 with no dropout, 0.743 at 10% dropout, 0.726 at 20%, 0.66 at 30%, 0.604 at 40%, 0.510 at 60%, and 0.317 at 80% dropout. These results suggest that higher dropout rates negatively impact ASW, indicating a decline in the ability to accurately cluster or differentiate cell types as data sparsity increases. Thus, ASW values are sensitive to dropout levels in scRNA-seq data, with lower dropout associated with more precise clustering. However, ZIGACL’s performance remained robust, demonstrating resilience to incomplete data and effectiveness in scRNA-seq data clustering.

ZIGACL’s runtime performance was evaluated across nine different datasets, showcasing variability in computational efficiency. The runtime for the Muraro dataset was 577 s, while for the Qx_Bladder dataset, it was 768 s. The Qx_Limb_Muscle dataset required 1028 s, and the Qx_Spleen dataset had the highest runtime at 1451 s. In contrast, the QS-seq2_Diaphragm dataset demonstrated the shortest runtime at 323 s. For the QS-seq2_Limb_Muscle and QS-seq2_Lung datasets, runtimes were 438 and 455 s, respectively. The Romanov dataset required 860 s, while the Klein dataset had a runtime of 469 s.

We used Seurat (https://github.com/satijalab/seurat) to compare the performance of different clustering methods across various datasets, including Muraro, Romanov, Klein, Qx_Bladder, Qx_Limb_Muscle, Qx_Spleen, QS-seq2_Diaphragm, QS-seq2_Limb_Muscle, and QS-seq2_Lung. The Adjusted Rand Index (ARI) scores for these datasets were 0.507, 0.420, 0.627, 0.495, 0.427, 0.251, 0.532, 0.432, and 0.360, respectively, while NMI scores were 0.746, 0.628, 0.720, 0.561, 0.706, 0.491, 0.746, 0.728, and 0.731. The average ARI and NMI scores for Seurat were ~ 0.450 and 0.673, respectively. ZIGACL demonstrated superior performance, achieving higher average scores for both metrics across all datasets. Specifically, ZIGACL improved the average ARI score by 0.296, representing a 65.8% increase, raising it from 0.450 to 0.746. For the NMI metric, ZIGACL showed an improvement of 0.116, representing a 17.2% increase, raising the score from 0.673 to 0.789. ZIGACL generally outperforms ScTAG [30] in several datasets, particularly in the Muraro and Qx_Limb_Muscle datasets, where it achieved ARIs of 0.912 and 0.989, respectively, exceeding ScTAG’s ARIs of 0.8878 and 0.9581. ZIGACL also showed improved NMI in these datasets. However, in other datasets like QS-seq2_Diaphragm and QS-seq2_Limb_Muscle, ZIGACL’s ARI and NMI scores were slightly lower, with an ARI of 0.947 compared to ScTAG’s 0.9628 in Diaphragm, and NMI of 0.752 compared to 0.9616 in Limb_Muscle. Overall, ZIGACL outperforms ScTAG in several cases but has some datasets where its performance is slightly lower. When compared to scGADR [31], ZIGACL improved ARI by 4.87%, increasing from 0.7114 to 0.746. Additionally, ZIGACL showed a 2.75% improvement in NMI, rising from 0.7679 to 0.789. Overall, ZIGACL demonstrated better performance in both ARI and NMI across all datasets.

While the ZIGACL method presents a novel and promising approach, several potential pitfalls merit consideration. Firstly, the complexity of combining these advanced models may lead to computational inefficiencies and challenges in implementation, particularly with large-scale datasets. Secondly, the dynamic adjustments in the learning process via a co-supervised deep graph clustering model may introduce variability and sensitivity to hyperparameter tuning, potentially affecting reproducibility and stability of results. Thirdly, while the constrained size of datasets may currently limit broad applicability, the anticipated future availability of larger single-cell datasets is expected to significantly enhance the model’s comprehensive validation and improvement. Additionally, the interpretability of the combined model’s outcomes might be hindered by the intricate interplay between ZINB and GAT components, complicating the extraction of clear biological insights.

## Methods

### Data processing

The nine scRNA-seq datasets from the Hemberg Lab (https://hemberg-lab.github.io/scRNA.seq.datasets/human/pancreas) provide comprehensive insights into various human tissues, emphasizing cellular heterogeneity and functions under different conditions (Table 1). These datasets include the Muraro Dataset for pancreatic cell atlas, Romanov Dataset for neural cell transcriptomics, and Klein Dataset for early human embryo development, each contributing valuable information on cell composition and gene expression. Additionally, the Qx and QS series, covering tissues like the bladder, limb muscle, spleen, diaphragm, and lung, offer detailed analyses of tissue-specific gene expression and cellular functions. To improve data quality in scRNA-seq datasets, genes not expressed in any cells were removed. After this filtering, normalization and scaling adjusted for sequencing depth and cell-specific effects. For each gene, the mean and variance of expression were computed, and a coefficient of dispersion was calculated as the variance-to-mean ratio. Genes were ranked by their adjusted dispersion values, and the top 2500 with the highest values were selected as high-variability genes, highlighting their potential biological significance. The adjacency matrix *A* was constructed using a Gaussian kernel function, which employed the cosine distance between cells *i* and *j* to quantify similarity. Specifically, the matrix element ${A}_{ij}$ was computed as


(1) \begin{equation*} {A}_{ij}=\exp \left(-\frac{{\left\Vert{d}_{ij}\right\Vert}^2}{2{r}^2}\right) \end{equation*}

where ${d}_{ij}$ represents the cosine distance between cells, and *r* is a tunable parameter that modulates the smoothness of the resulting function.

### ZINB autoencoder for addressing the sparsity and over-dispersion of scRNA-seq data

The core architecture of autoencoder consists of an encoder and a decoder. The encoder function transforms the normalized input, ${X}_{z- scor\mathrm{e}}$, which includes 2500 high-variability genes, into a compact latent representation denoted as *H*. Subsequently, the decoder attempts to reconstruct *H* back into the approximated input data, $\overline{X}$. The encoder is structured with *L* layers, and the representation from the $l$-*th* layer is expressed as ${H}_e^l$. This layer-specific representation is derived using associated weights ${w}^l$ and bias vectors ${b}^l$. The adaptive learning process for each layer, particularly the $l$-*th* layer, is intricately defined to optimize the transformation from high-dimensional data to a meaningful encoded space.


(2) \begin{equation*} {H}_e^l=\varnothing \left({w}^l{H}_e^{\left(l-1\right)}+{b}^l\right) \end{equation*}

The encoder stage maps $ {X}_{z- scor\mathrm{e}}$ to the latent representation ${H}_e$ as:


(3) \begin{equation*} {H}_e={f}_{enc}\left(W{X}_{z- scor\mathrm{e}}+b\right) \end{equation*}

Instead, the decoder stage takes as input the latent representation ${H}_e$ and produces a reconstruction $\overline{\mathrm{X}}$ of the original input ${X}_{z- scor\mathrm{e}}$:


(4) \begin{equation*} \overline{\mathrm{X}}={f}_{dec}\left({W}^{\prime }{H}_e+{b}^{\prime }\ \right) \end{equation*}

where *W* and ${W}^{\prime }$ denote the weight matrices for the encoder and decoder respectively, while *b* and ${b}^{\prime }$ correspond to their bias vectors.

The encoder and decoder are formulated as neural networks, specifically as multilayer perceptions (MLP). To compute loss probability *π*, the mean *μ*, and dispersion *θ*, three distinct fully connected layers are employed. These are expressed mathematically as:


(5) \begin{equation*} D={f}_{dec}\left({f}_{enc}\left({X}_{z- scor\mathrm{e}}\right)\right) \end{equation*}

(6) \begin{equation*} \pi = Sigmoid\left({W}_{\pi }D\right) \end{equation*}

(7) \begin{equation*} \mu = Diag\left[\mathit{\exp}\left({W}_{\mu }D\right)\right] \end{equation*}

(8) \begin{equation*} \theta =\mathit{\exp}\left({W}_{\theta }D\right) \end{equation*}

In this setup, *D* represents the output matrix from the decoder’s final layer. Given that the loss probability π ranges between 0 and 1, a sigmoid function is utilized for normalization purposes. Considering that both mean and dispersion are inherently non-negative, the exponential function is aptly selected as the activation function. To mitigate overfitting, normalization of the mean μ is applied, aligning it with the original count size for each cell through a diagonal operation. The weights ${W}_{\pi }$, ${W}_{\mu }$, and ${W}_{\theta }$ are defined as trainable parameters within the model. The loss function is articulated as the sum of the negative logarithms associated with the ZINB distribution as follows:


(9) \begin{equation*} {L}_{ZINB}=\sum -\log \left( ZINB\left({E}_{n\times m}^{\prime }|\pi, \mu, \theta \right)\right) \end{equation*}

Our unsupervised autoencoder approach, which incorporates the Zero-Inflated Negative Binomial distribution, effectively captures the sparsity and over-dispersion typical of scRNA-seq data, enabling robust simulation and the extraction of meaningful biological insights from these datasets. The ZINB is formulated as


(10) \begin{equation*} ZINB\left({E}_{n\times m}^{\prime }|\pi, \mu, \theta \right)=\pi \times \delta \left({E}_{n\times m}^{\prime}\right)+\left(1-\mathrm{\pi} \right)\times NB\left({E}_{n\times m}^{\prime }|\ \mu, \theta \right) \end{equation*}

where


(11) \begin{equation*} NB\left({E}_{n\times m}^{\prime }|\mu, \theta \right)=\frac{\Gamma \left({E}_{n\times m}^{\prime }+\theta \right)}{\Gamma \left({E}_{n\times m}^{\prime }+1\right)\Gamma \left(\theta \right)}{\left(\frac{\theta }{\theta +\mu}\right)}^{\theta }{\left(\frac{\mu }{\theta +\mu}\right)}^X \end{equation*}

In our model, $E{\prime}_{n\times m}$ represents the original count matrix of gene expressions with 2500 high-variability genes, where *n* is the number of cells and *m* is the number of genes. The negative binomial component, denoted as NB, is parameterized by the mean $\mu$ and dispersion $\theta$, while $\pi$ indicates the probability of dropout events, modeling the zero-inflation aspect. Furthermore, $\varGamma$ represents the Gamma distribution, while $X$ denotes the actual observed value of the random variable that follows the Negative Binomial distribution. The probability mass function of the ZINB quantifies the likelihood of observing zero counts, effectively distinguishing between biological zeros and technical dropouts in gene expression data.

### GAT for identifying structural features of scRNA-seq data

To tackle the challenges of scarce structural data in feature extraction using an autoencoder, we employ the GAT. The GAT’s graph attention layer dynamically adjusts node representations by aggregating and weighting data from neighboring nodes. Utilizing a normalized gene expression matrix and an adjacency matrix, we integrate outputs from both the autoencoder and GAT, achieving a representation that captures gene expression and cell topology.

In the graph’s attention layer $l$, the representation ${z}_i^{(l)}$ for cell *i* is calculated as follows:


(12) \begin{equation*} {z}_i^{(l)}=\sigma \left(\sum_{j\in{N}_i}{\alpha}_{ij}^l{W}^l{z}_j^{\left(l-1\right)}\right) \end{equation*}

where ${z}_i^{(l)}$ signifies the hidden layer representation, ${N}_i$ denotes the set of neighboring cells, ${a}_{ij}$ represents the attention coefficient illustrating the relevance of cell *j* to cell *i*, and ${W}^l$ represents a learnable parameter matrix for graph attention layer $l$. The activation function *σ* (·) can be specified as *LeakyReLU*.

From a topological standpoint, adjacent nodes exert influence on the target node via connecting edges. Unlike classical GATs that consider only first-order neighborhoods, our graph attention layer accounts for higher-order neighborhoods. To this end, we introduce a nearest neighbor matrix *R* to quantify the topological correlation among high-order neighboring nodes, as described in (13):


(13) \begin{equation*} R=\left(B+{B}^2+\dots +{B}^t\right)/t \end{equation*}

where *B* is the transition matrix. If there is an edge between nodes ${v}_i$ and ${v}_j$, then ${B}_{ij}=1/{m}_i$; otherwise, ${B}_{ij}=0$. Here, ${m}_i$ denotes the degree of node ${v}_i$, and ${R}_{ij}$ signifies the topological correlation between nodes *i* and *j* up to order *t*. The attention coefficient ${\alpha}_{ij}$, incorporating both topological weights and activation functions, is defined as follows:


(14) \begin{equation*} {\alpha}_{ij}=\frac{\exp \left({R}_{ij}\left( LeakyReLU\left({c}_{ij}\right)\right)\right)}{\sum_{r\in{N}_i}\exp \left({R}_{ij}\left( LeakyReLU\left({c}_{ij}\right)\right)\right)} \end{equation*}

To calculate ${c}_{ij}$, we explore the relationship between cell *i* and cell *j* via a linear transformation,


(15) \begin{equation*} {c}_{ij}={a}^T\left[W{x}_i\parallel W{x}_j\right] \end{equation*}

where $a$ is a weight vector, $\parallel$ is a connection operation, ${x}_i$ and ${x}_j$ are the input features of cell *i* and cell *j*.

Next, we balance the parameter λ to blend the representation obtained from a ZINB autoencoder with the graph attention layer at each level. The resulting fusion representation ${\overline{z}}^{\left(l-1\right)}$ is derived as follows:


(16) \begin{equation*} {\overline{z}}^{\left(l-1\right)}=\left(1-\lambda \right){z}^{\left(l-1\right)}+\lambda{H}_e^{\left(l-1\right)} \end{equation*}

where ${z}^{\left(l-1\right)}$ denotes the output of the $l-1$ level in GAT, and ${H}_e^{\left(l-1\right)}$ is the output from the same level in the autoencoder. By combining these two representations at each layer, we achieve a balanced representation. This fusion output, ${\overline{z}}^{\left(l-1\right)}$, then serves as the input for the *l-th* layer of the GAT, as shown in the following expression:


(17) \begin{equation*} {z}^{(l)}=\sigma \left(\ \sum_{j\in{N}_i}{\alpha}_{ij}^l{W}^l{\overline{z}}^{\left(l-1\right)}\right) \end{equation*}

### Co-supervised learning

Integrating an encoding layer from an autoencoder into a GAT module enriches feature representation within the latent space but presents challenges for immediate clustering. To address this, leveraging co-supervised learning effectively trains the complete network, refining subsequent clustering tasks by using synergies between feature extraction and data organization.

For a given cell *i* and cluster *k*, a soft label ${q}_{ik}\in Q$ is defined, signifying the similarity between the data representation ${h}_i$ of the *i-th* encoding layer and the cluster center vector ${u}_k$, which is initialized through K-means clustering. Here, $Q$ denotes the set of probabilities that all cells are attributed to cluster centers. In determining a probability distribution model, the Student’s t-distribution is employed to quantify the similarity between ${h}_i$ and ${u}_k$:


(18) \begin{equation*} {q}_{ik}=\frac{{\left(1+{\left\Vert{h}_i-{u}_k\right\Vert}^2/v\right)}^{-\frac{v+1}{2}}}{\sum_{k^{\prime }}{\left(1+{\left\Vert{h}_i-{u}_{k\prime}\right\Vert}^2/v\right)}^{-\frac{v+1}{2}}} \end{equation*}

where $v$ represents the degree of freedom of the Student’s t distribution. The similarity ${q}_{ik}$ is also known as soft cluster assignment, which is viewed as the probability of assigning cell *i* to cluster center $k$. ${k}^{\prime }$ is an index iterated over all cluster centers, used to normalize the membership degree of data point ${h}_i$ for each cluster center ${u}_k$. In this process, the data is expected to be as close as possible to the cluster center of the real data. Therefore, we use the soft label frequency ${F}_k$ to obtain a higher confidence data representation ${p}_{ik}\in P$.


(19) \begin{equation*} {F}_k=\sum_i{q}_{ik} \end{equation*}

(20) \begin{equation*} {p}_{ik}=\frac{{q_{ik}}^2/{F}_k}{\sum_{k^{\prime }}{q_{i{k}^{\prime}}}^2/{F}_{k^{\prime }}} \end{equation*}

where ${q}_{ik}$ is squared and normalized in order to bring the feature representation closer to the cluster center. Finally, KL divergence is adopted, making the soft clustering assignment close to the target assignment. The clustering loss is as follows:


(21) \begin{equation*} {L}_{clu}= KL\left(P\parallel Q\right)=\sum_i\sum_k{p}_{ik}\log \frac{p_{ik}}{q_{ik}} \end{equation*}

By minimizing the KL divergence loss between the Q distribution and the P distribution, the target distribution P can help the autoencoder module better learn the representation of the clustering task by pulling the data closer to the cluster center.

Likewise, to train a graph attention neural network, the KL divergence loss is as follows:


(22) \begin{equation*} {L}_{GAT}= KL\left(P\parallel Z\right)=\sum_i\sum_k{p}_{ik}\log \frac{p_{ik}}{z_{ik}} \end{equation*}

where the feature matrix $Z$, derived from training the GAT, contains cellular characteristics from the inter-cellular relationships and the associations between cells and gene expressions. The primary goal of both the ZINB autoencoder and GAT module is to accurately approximate the target distribution $P$, thereby enabling mutual supervision of the learning processes. The optimization endeavors include the reconstruction loss ${L}_{ZINB}$, clustering learning loss ${L}_{clu}$, and the classification loss ${L}_{GAT}$. Consequently, the ultimate loss function is defined as follows:


(23) \begin{equation*} L={L}_{ZINB}+{\alpha L}_{clu}+{\beta L}_{GAT} \end{equation*}

In this study, $\alpha$ serves to equilibrate the dual objectives of cluster optimization and the preservation of local structure, and is preset to a value of 0.1. $\beta$ modulates the interaction of the GAT with the embedding space, with its default setting at 0.01.

### Competing methods

Advanced computational techniques have significantly enhanced scRNA-seq analysis, improving clustering accuracy and cell type identification. scGNN utilizes a graph neural network to capture complex cell relationships through graph-based representation, enhancing gene expression detection [16]. DESC uses a deep learning framework with stochastic optimization to increase clustering robustness and reproducibility [17]. SC3 combines multiple unsupervised learning methods to provide robust and consistent clustering by aggregating various outcomes [18]. The Leiden algorithm optimizes detection of small cell communities in k-nearest neighbor graphs, enhancing clustering granularity [19]. scDeepCluster merges deep learning with clustering via an autoencoder and clustering layer, aiding in the discovery of novel cell types [20].Graph-sc [21] and Scanpy [22] offer advanced tools for maintaining local and global cellular structures and scalable single-cell gene expression analysis respectively.

### Elevation metrics

The clustering effectiveness of datasets labeled with cell types is evaluated using the Adjusted Rand Index (ARI) and Normalized Mutual Information (NMI) [32]. The ARI measures the similarity between the clustering labels predicted by the algorithm and the reference clustering labels, and is expressed as:


(24) \begin{equation*} ARI=\frac{\sum_{ij}\left(\genfrac{}{}{0pt}{}{n_{ij}}{2}\right)-\frac{\left[\sum_i\left(\genfrac{}{}{0pt}{}{a_i}{2}\right)\sum_j\left(\genfrac{}{}{0pt}{}{b_j}{2}\right)\right]}{\left(\genfrac{}{}{0pt}{}{n}{2}\right)}}{\frac{1}{2}\left[\sum_i\left(\genfrac{}{}{0pt}{}{a_i}{2}\right)+\sum_j\left(\genfrac{}{}{0pt}{}{b_j}{2}\right)\right]-\frac{\left[\sum_i\left(\genfrac{}{}{0pt}{}{a_i}{2}\right)\sum_j\left(\genfrac{}{}{0pt}{}{b_j}{2}\right)\right]}{\left(\genfrac{}{}{0pt}{}{n}{2}\right)}} \end{equation*}

where ${n}_i$ represents the number of cells shared between cluster *i* in the true labels and cluster *j* in the clustering result, *n* is the total number of cells, with ${a}_i=\sum_j{n}_{ij}$ and ${b}_j=\sum_i{n}_{ij}$ respectively.

The NMI clustering metric is used to assess the quality of clustering results. It quantifies the mutual information between the predicted clustering labels and the reference clustering labels, expressed as:


(25) \begin{equation*} NMI=\frac{MI\left(P,T\right)}{\sqrt{H(P)\ H(T)}} \end{equation*}

where *T* represents the true labels of the sample points, *P* denotes the clustering results, *MI(·,·)* symbolizes mutual information, and *H(·)* represents the entropy function. An increase in NMI value indicates stronger consistency between the two clusters, suggesting more robust and consistent overlap in data distribution.

The Silhouette Coefficient, ranging from −1 to 1, assesses clustering effectiveness. It measures similarity within clusters versus separation from others using the formula


(26) \begin{equation*} s=\frac{b-a}{\max \left(a,b\right)} \end{equation*}

where *a* is the average intra-cluster distance (cohesion), and *b* is the distance to the nearest different cluster (separation). Values close to 1 indicate compact, well-separated clusters, while values near −1 suggest poor clustering with misplaced samples.

**Key Points**

- ZIGACL combines a Zero-Inflated Negative Binomial model with a Graph Attention Network, leveraging mutual information from neighboring cells and integrating denoising and topological embedding to enhance clustering accuracy.
- ZIGACL enhances cell clustering across different scales of single-cell data, effectively addressing scalability challenges.
- ZIGACL addresses sparsity and dropout in single-cell RNA-seq by using co-supervised deep graph clustering for better denoising and accuracy.

## Code availability

The ZIGACL algorithm implemented in Python and is available on GitHub (https://github.com/ShiMGLab/ZIGACL).
